# Assessing Knowledge and Perceptions About Cancer Among American Indians of the Zuni Pueblo, NM

**DOI:** 10.1007/s13187-021-02023-0

**Published:** 2021-05-07

**Authors:** Safia Safi, Donica Ghahate, Jeanette Bobelu, Andrew L. Sussman, Joseph Rodman, Angela Wandinger-Ness, Shiraz I. Mishra, Thomas Faber, Cheryl Willman, Vallabh Shah

**Affiliations:** 1 Department of Internal Medicine, University of New Mexico Health Sciences Center, Albuquerque, NM, USA; 2 Department of Family and Community Medicine, University of New Mexico Health Sciences Center, Albuquerque, NM, USA; 3 University of New Mexico Comprehensive Cancer Center, Albuquerque, NM, USA; 4 Department of Pathology, University of New Mexico Health Sciences Center, Albuquerque, NM, USA; 5 Department of Pediatrics, University of New Mexico Health Sciences Center, Albuquerque, NM, USA; 6 Indian Health Service, Zuni Comprehensive Care Center, Zuni, NM, USA; 7 Department of Biochemistry and Molecular Biology, School of Medicine, University of New Mexico, MSC 08 4670, Albuquerque, NM 87131, USA

**Keywords:** American Indians, Cancer knowledge, Cancer perception, Community Health Representatives

## Abstract

American Indians (AIs) in New Mexico have lower cancer screening rates compared to other populations and are more likely to be diagnosed with cancer at an advanced stage of the disease as reported by Li et al. (*Archives of Internal Medicine* 163(1):49–56, 2003). AIs also have the lowest 5-year cancer survival rates compared to any ethnic/racial group in the USA as reported by Clegg et al. (*Arch Intern Med* 162:1985–1993, 2002) and Edwards et al. (*Cancer* 97:1407–1427, 2005). Numerous barriers such as cultural beliefs, fear, fatalism, mistrust, stigma, and lack of culturally appropriate interventions could contribute to low cancer screening rates as reported by Daley et al. (*J Health Dispar Res Pract* 5(2), 2012); Filippi et al. (*J Prim Care Community Health* 4(3):160–166, 2013); James et al. (*Prev Chronic Dis* 10:E170, 2013); and Schumacher et al. (*Cancer Causes Control* 19(7):725–737, 2008). Trained Community Health Representatives (CHRs) from the Zuni Pueblo and native Zuni undergraduate students led six 1-h focus group sessions using a structured focus group guide with probes. The focus groups were conducted among 51 participants from different age groups (20–29 years, *n* = 19; 30–49 years, *n* = 17; and 50 years and older, *n* = 15) stratified by sex. Focus groups were conducted in both English and Shiwi (Zuni) languages. Sessions were audio recorded, and team members took notes. CHRs transcribed the notes and audio recordings, and created a codebook for qualitative data analysis. In the focus groups, participants provided Zuni-specific cultural context, opinion, and experience regarding (1) general knowledge about cancer, (2) cancer risk, (3) cancer risk reduction, (4) personal experiences with cancer, and (5) culturally competent delivery of cancer information and resources. Understanding the perceptions of cancer within the Zuni Pueblo is an essential component in the development of interventional/preventative measures and improvement of current care. Ultimately, this information will provide a basis for the next steps in culturally sensitive cancer care for the Zuni Pueblo.

## Introduction

Cancer is a major health concern among American Indian/Alaska Native (AI/AN) populations. In comparison to other populations, AI/AN have lower cancer screening rates, are diagnosed at late stage of the disease, and are less likely to receive treatment [1]. Malignant neoplasm is the second leading cause of death in AI/AN [2]. For several cancers, there are still persistent disparities among AI/AN populations [3]. Within the USA, AI/AN have some of the lowest 5-year cancer survival rates [3]. Cancer is a stigmatized disease in American Indian (AI) culture due to its association with death [4, 5]. This has resulted in a decreased willingness for many members of indigenous communities to engage in open conversations about the disease and has contributed to low levels of knowledge about its etiology, diagnosis, and treatment opportunities. [4, 5].

The Zuni Pueblo, located in western New Mexico, is home to a rural AI community. They have lived in the American Southwest for thousands of years [6]. The Zuni tribe is unique in that it differs culturally and ethnically from neighboring tribes [7]. It is considered an endogamous population due to low levels of emigration and immigration [8]. Many of the tribe’s people rely on their traditional income from horticulture, jewelry making, painting, and pottery [6]. The community has suffered from historical trauma and high rates of poverty, which has affected health outcomes and health literacy within the Pueblo [9]. Such disparities in turn affect cancer control outcomes in Zuni, where cancer screening rates remain low.

### The Cultural Context

When addressing cancer control disparities in Zuni and other AI communities, it is essential to consider barriers to health that are a result of a specific cultural context that generate and perpetuate specific cultural beliefs. Barriers that contribute to low cancer screening rates and survivorship include cultural beliefs in health and health care, fear, fatalism, mistrust, stigma, and lack of culturally appropriate interventions [10-12]. Such barriers foster a strong stigma against discussions about diseases including cancer in AI families across many of the tribal communities in New Mexico. As a result, cancer screening and treatment programs offered by institutions, including Indian Health Services and local cancer centers, have faced barriers in their outreach efforts. This pervasive stigma has also contributed to the fact that AIs are diagnosed with cancer at a higher rate compared to other ethno-racial groups, despite intensive risk-reduction programs.

Contextual factors are important when examining health behaviors [13]. This theoretical foundation informs our participatory study approach that places community interests at the core of the definition of the problem [13]. The aim of this study was to employ a culturally centered qualitative study design to examine the knowledge, opinions, and experiences that the Zuni people have about the nature of cancer and how these perspectives can inform the design, development, and dissemination of effective culturally appropriate interventions [14].

## Methods

### Overall Design

In order to address the need for a contextualized or culturally informed knowledge, we conducted a qualitative study designed to probe the specific culture-bound knowledge and understanding Zuni Indians have about cancer and the disease process of cancer with effect of health behaviors and nutrition [15]. We convened a set of focus groups to respond to questions about cancer within the specific context of the Zuni community and culture.

### Recruitment

This study was approved by the University of New Mexico Health Sciences Center Human Research Review Committee. The research team also gained approval from the Zuni tribal council for this research prior to funding. Furthermore, the principal investigator (VOS) maintains a memorandum of understanding with the Zuni tribe outlining conditions for all publications. This manuscript has been shared with the tribal leaders and stakeholders.

All participants provided written consent and received $25 as appreciation for sharing their time and expertise. Potential study participants were recruited using two methods. First, Zuni Community Health Representatives (CHRs) used the Zuni Health Initiative (ZHI) project’s clinical database, which contained records on 25% of the Zuni community, to identify eligible tribal members. Second, individuals were recruited through visits by CHRs to Zuni households, presentations at tribal health programs and at the health care center, distribution of flyers at local businesses and the civic center, and through other health programs. Given the exploratory focus of the study, inclusion criteria were broad and included all tribal members over the age of 18.

### Focus Group Data Collection

The focus group sessions were conducted at the ZHI office between April 6, 2018, and May 16, 2018. The group sessions were facilitated in both English and Shiwi (Zuni language) by a CHR or native Zuni undergraduate students. The CHRs used in this study were trained in qualitative studies and have over 20 years of experience. CHRs and the principal investigator (VOS) trained Zuni undergraduates in focus group methodology. Each of the focus groups was audio recorded and supplemented with note taking to ensure accurate transcription of the sessions. Through the written consent and overview of focus group procedures, participants were made aware that they would be audio recorded during the focus groups and were provided the opportunity to decline participation if they were not comfortable. A focus group guide was developed by the principal investigator (VOS) and reviewed by study team staff. It consisted of eight questions that were complemented by probes to help facilitate dialog (Table 1). The focus group participants were asked open-ended questions pertaining to the following cancer-focused domains: knowledge, risk, risk reduction, personal experiences, and culturally competent delivery of cancer information. In this way, the focus group guide standardized the questions being asked in each session. Follow-up questions and probes helped further facilitate discussion among focus group participants. The focus group questions were designed to facilitate active discussions among the participants in order to uncover perceptions of cancer in the specific cultural context of the Zuni tribal community.

### Data Analysis

Once focus group recording and note taking was complete, trained Shiwi-speaking ZHI staff transcribed the recordings and made observational notes. Transcripts were then analyzed using a three-phase iterative approach to achieve data saturation. First, analysts read each of the six focus group transcripts independently to gain a baseline understanding and familiarity with the material and generate an initial set of thematic codes. Next, analysts worked together to review these summaries and refine the coding template. Once finalized, the coding template was applied to NVivo, a qualitative software analysis program, to support detailed assessment of thematic variation and facilitate data queries. ZHI staff reviewed data report summaries to assess cultural factors not readily apparent to non-Zuni members of the research team. Lastly, analysts generated narrative summaries by reviewing the master spreadsheet of observations produced in the previous phase and also referring back to the original transcripts when further exploration of source material was necessary. This iterative process enabled the team to determine data saturation had been reached when it was evident no new thematic categories emerged in later stages of data collection. For each narrative summary, analysts extracted exemplary participant responses into discrete categories or themes for reporting purposes. Further description of data analysis procedures is included in Table 2.

## Results

Participants in the focus groups were comprised of distinct demographic segments of the Zuni Pueblo. Community members were recruited to join one of six 1-h focus group discussions. A total of 51 community members participated in the focus groups, comprised of separate male and female cohorts for the following age groups: 20–29 years (*n* = 19), 30–49 years (*n* = 17), and 50 years and older (*n* = 15), as listed in Table 3. Individuals were not required to have direct experience with cancer, although many did. We have organized the presentation of findings aligned with the major domains of the interview guide. The results represent consensus responses from the focus groups. We identify divergent and distinctive perspectives when relevant.

### General Knowledge of Cancer

Responses regarding general knowledge of cancer can be grouped into three distinct categories: (1) a general lack of knowledge, (2) the perception that the disease is uncontrollable in nature, and (3) negative connotations about cancer.

Participants acknowledged not knowing much about the disease. Some expressed their desire to learn more about cancer as their motivation for participation in the focus group.

I don’t really know much about cancer so that’s why I wanna know more about it, so that’s why I’m here.

(Male 30–49)

Participants expressed uncertainty about the different factors that contribute to cancer:

I’m a [cancer] survivor… I don’t know where it came from.

(Female 50+)

These responses suggested some ambiguity, inconsistency, or uncertainty about the causes of cancer among some local community members.

I don’t really know much about it, how you get, you know, like I said… people who don’t smoke get it…

(Male 50+)

Many participants saw cancer as something uncontrollable in nature and that may eventually lead to death. One participant’s description of cancer follows:

Well, to me, it’s scary and I don’t know how you get it but probably when you go to the hospital and they find out that you have one, and when they tell you it’s scary cause you might die or I may, I might survive. Stuff like that, so I’m scared of that one.

(Female 50+)

Others described the irrepressible nature of cancer in more of a clinical context, through their own lived experience: For instance, one man said:

You can never, well, never say never, but you can cure it but then it’ll come back. I know it because my grandma had cancer and she cured it, but it obviously came back and it killed her so you can’t cure cancer.

(Male 20–29)

Participants from all groups expressed negative connotations when they heard the word “Cancer.” For instance:

…I think that is the […] scariest word when you think about your health.

(Male 50+)

Similarly, participants linked an adverse association with the word “cancer” to their own personal experiences:

When I hear cancer it’s heartbreaking because I had a loved one who died from cancer.

(Female 50–75)

### Cancer Risk

Participant attributions of cancer risk fell into three general categories, largely adhering to “textbook” explanations about the causes of cancer: (1) lifestyle activities/choices, (2) environmental and workplace exposures, and (3) heredity.

Lifestyle activities included smoking tobacco, drinking alcohol, eating processed food, and exercise (how people “take care of themselves”). Participants conveyed the idea that personal choice can dictate whether individuals get cancer or not.

… there’s a lot of processed foods out there so you know… there’s not a lot of organic things around here so it’s, it’s very hard. I have… somebody who’s had… a tumor and he had to go to radiation and chemo and therapy and all that. But he found out that that stuff was getting him sick so… he got himself off it, which the doctors and everybody else didn’t like… cause that what they get money off of… the chemo and everything else; all the treatments and everything so… he… didn’t agree with them. He fought for it and right now he’s sticking to everything organic, which turned to plates, forks, cups. Everything organic. And right now it’s been over a year since he’s been free from cancer, so I believe it’s a yes and no situation, you know. You just have to take care of yourself… there’s a lot of risk with everything around outside, inside so it’s just pretty much taking care of yourself.

(Female 20–29)

Environmental and workplace causes of cancer included exposure to chemicals, asbestos, and other materials perceived to be unhealthy. Participants suggested that inhalation of dust from traditional employment in carving rocks or other materials may be a cause of cancer. One participant elaborates:

Most Zunis are silversmith and fetish making and all that grinding dust and buffing our jewelry… all that inhaling of the powder and dust I think that’s what most of us Zunis… are at greater risk of having cancer.

(Male 50+)

Lastly, respondents also identified the hereditary nature of cancer. These discussions highlighted their view of the opaque nature of the influence of a family history of cancer in comparison to environmental and other behavioral risk factors. One respondent said:

It’s possible [if someone else in my family had it that I can get it] but you can probably if, but god forbid you do get it, there is a way to find out early on is why they teach us how to feel for bumps and get that checked out right away so yeah.

(Female 50+)

Overall, focus group participants expressed an understanding that a family history of cancer increases risk of developing some type of cancer.

I think the one other thing that make people more susceptible… was genetics. So some people have a higher genetic predisposition than others…

(Female 20–29)

Collectively, participants’ view of environmental and workplace exposures and heredity suggest causes of cancer that are often beyond what individuals can control through their personal choices. For instance, one man ruminated about his professional experience renovating homes:

I think it’s through the dust on what you carve and what kind of rock you use… that alabaster is the one that can… get you sick… it’s all types of rocks. Some of those old houses that… I use to do…home renovations before… some of these old houses that we renovated had asbestos paneling up in the ceilings and sheetrock. None of us back then, none of us didn’t know anything about covering ourselves up. We were in there with our mouths open… nose uncovered… short sleeves… none of us took any preventions…

(Male 30–49)

Participants across all six focus groups expressed the belief that everyone is at risk for getting cancer. It was commonly understood that cancer affects individuals of all age, race/ethnicity, sex, and social/economic classes.

### Cancer Risk Reduction

The themes that emerged from discussions of lowering individual risk of cancer are as follows: (1) everyone can get cancer and it is important to be screened and (2) the importance of reducing risk through behavioral changes including diet, exercise, and other cancer prevention efforts including screening and other interactions with the health care system.

One participant synthesized both of these themes, highlighting the perspective that everyone is at risk for cancer, behavior and genetics affect risk, and screening and knowledge of cancer are important:

Somebody was really fit still can be at risk and I believe that it’s more about the genetics because exercising more it’s basically more for different kind of disease that will help prevent but for cancer I don’t believe that, that maybe yeah exercising regularly will help prevent cancer is more like just yeah early detection and just be more knowledge about what cancer’s all about and it’s you can learn more.

(Male 20–29)

Another participant focused on the behavioral choices one can make to reduce risk:

Just overall good healthy diet I guess if you can afford it, exercise, just all be aware of what your intake is and how you feel and you just, if you’re not feeling good of course, go get checked, which is one way to find out what’s going on with you.

(Male 30–49)

### Personal Experiences with Cancer

In every focus group, respondents shared experiences of family members (or selves) who experienced cancer. These responses ranged from brief descriptions of a diagnosis and treatment process to lengthier and sometimes emotional stories of a family member navigating through the care process and succumbing to the illness. These reflections can be grouped into two thematic categories: (1) confusion and uncertainty, both in terms of understanding the cause of their family member’s cancer and navigating and making sense of the treatment process, and (2) the emotional challenges of supporting and caring for these loved ones with cancer (including themselves), coping with suffering, and, in some cases, death. One respondent described this sense of disorientation:

I wish I knew what I could do or wish I know how the drug that could take care of it but we don’t know, we all don’t know anything. What cancer not, not that well even the doctors don’t know that well about cancer, different types of cancers what, whatever cancer their trying to cure, they don’t really. They have an idea but they really don’t know they just write, use all kinds of drugs to help you, help you cure it or make you comfortable

(Male 30–49)

Another participant reflected on the emotional expense of a cancer diagnosis for a family member:

She was a special person to a lot of people, it didn’t just affect my family. She knew all kinds of people and when she passed a lot of people didn’t know how to deal with it, she was still young. There’s not really a way to cope with it but just to let it out, talk about it with somebody - doesn’t matter how it looks. There really is no way to get over it. It’s still gonna be with me, but doesn’t really go away. There is really no way to cope with it.

(Female 30–49)

Another participant spoke about the prospect of her own cancer diagnosis:

I think one of the things that I deal with is if it were to happen to me, what would I do? How would I feel? Who will be there? I don’t like to talk about it, but I think the way to educate yourself is to face your fears and just get into learning about things.

(Female 30–49)

Throughout the focus group discussions, participants noted the importance of providing support for community and family members. It was clear that those focus group participants who cared for family members with cancer suffered and grieved. These feelings persisted even after the person passed. For some, these emotions were further complicated by a struggle to understand the cause of the cancer diagnosis, and the challenge of making sense of the treatment process. These experiences led to frustration and fear. This aligns with themes from these focus groups touched on else-where here in which participants express confusion about the causes of cancer and the inscrutable, uncontrollable notion that everyone is at risk regardless of behavior or heredity.

### Culturally Competent Delivery of Cancer Information and Resources

Focus group participants were asked two questions to better understand how to effectively deliver to the Zuni community cancer information and resources that may result in increased screening rates. These comprised as follows: (1) What kind of information is best to learn about cancer? and (2) Are there any programs or resources that you would like to see in the community to help people who may have cancer?

Participants suggested three platforms that provide the best settings to learn about cancer: (1) group sessions, (2) one-on-one sessions, and (3) traditional (print) media. The consensus among participants identifies an overall lack of knowledge about cancer among community members and more information, in a variety of forms, would be helpful to address this gap, and ultimately increase screening. Responses to this question highlight some of the barriers to health Zuni community members perceive. For instance, one participant observed:

… the community here isn’t so social… a lot of people that don’t like to talk about anything… they’ve got negative feelings about what’s going to happen if they were to say something or be a part of something and… it’s hard to get people to go to a group session… I think that instead of expecting the community to come forward and participate it should be more or less be putting more information out there and… maybe pamphlets, not… only at the hospital but at different places; posters, phone numbers they can call, people they can contact…

(Female 30–49)

Participants noted that talking about cancer in the community can be considered taboo. They broadly agreed that more information should be made available to the community so members can better understand and cope with cancer.

Secondly, there was no mention of a specific program to address community needs; however, four potential strategies to distribute relevant information about cancer emerged from the discussions: (1) desire for group discussions, (2) need to raise awareness, (3) promote community involvement, and (4) provide treatment resources. Generally, the community members shared similar viewpoints about resources that could benefit the community as a whole. Most often, respondents offered group discussion and one-on-one discussion as effective venues to learn about cancer and share experiences. One participant explained:

Meetings like this, discussing, sharing information. Meetings like this can really help; hopefully, if they do start that, the people will understand and come together.

(Female 50+)

Some lamented the absence of treatment resources close to home. Indeed, Zuni community members have to travel far outside the pueblo in order to receive most cancer treatments. As one member said, “They need their own chemotherapy here.” (Male 20–29). More generally, participants acknowledged the importance of sharing experiences about cancer to help remind each other, as one participant put it, “that we’re not alone.” (Male 30–49).

## Discussion

This focus group study demonstrates how the Zuni people conceptualize cancer. Although cancer is a feature of Zuni lives, we identified culturally mediated knowledge gaps and discomfort with regard to discussing cancer. These factors likely contribute to noted cancer screening and treatment disparities among Zuni community members by preventing or discouraging them from taking active steps to address cancer through information- and care-seeking. Paradoxically, Zuni participants in these focus groups were generally aware and articulate about this outlook. Indeed, they frequently expressed a desire to learn more about cancer. These results show that there is both the need and desire within this community for additional culturally appropriate educational materials that emphasize the importance of having services to address emotional and health-related barriers.

A common thread throughout focus group discussions was the uncontrollable, unpredictable, and inevitably fatal nature of cancer. This view is not unique to Zuni; previous research among the Navajo echoes similar perceptions about cancer [3]. The notion that people can be ‘in two minds’ about cancer has been noted by Robb et al. [16]. A rapid, intuitive sense of dread and imminent death coexists with a deliberative, rational recognition that cancer can be a manageable, or even curable, disease. Their study found that despite recognition of improvements in outcomes, visceral fear of the disease persist and people have to struggle to control it [16]. Current understanding about fatalistic beliefs held by racial/ethnic minorities affect knowledge about cancer risk factors and knowledge of recommendations for cancer prevention [17]. Recognizing this perception of cancer and its final outcome is important to understand why it is not widely talked about within the community. Addressing the underlying fear of cancer can help to facilitate discussion in a culturally sensitive manner. Indeed, as suggested by Davis et al., it is essential to identify knowledge gaps pertaining to cancer risk factors in order to appropriately align educational and behavioral interventions [18]. Culturally relevant health education/promotion interventions need to be developed and tailored to (1) empower Zuni Indians regarding their ability to prevent cancer and (2) globally educate Zuni Indians about their susceptibility and risk perception for cancer.

## Conclusion

We collaborated with Zuni community members to contextualize their views of cancer and the many factors associated with it. From these views emerged many themes offering us the opportunity to address community cancer control needs. Understanding the perceptions of cancer within the Zuni Pueblo is an essential component in the development of interventional/preventative measures and improvement of current care. This study directly addresses the limited research on perceptions of cancer among AI/AN. The findings presented here may aid in the development of future interventions to decrease cancer health inequalities in AI/ AN communities. Ultimately, this information will provide a basis for the next steps in culturally sensitive cancer care for the Zuni Pueblo.

## Figures and Tables

**Table 1:** Focus group questions and probes

#	Question	Probe(s)
1	We would like to start by having you share what comes to mind when you hear to word “cancer.”	How would you define cancer? How would these definitions apply to different types of cancer, such as breast, cervical, or prostate?
2	We are also interested in your views about what might cause cancer. whether or not someone gets cancer?	What kinds of things have you heard about that might influence
3	What are your understandings about who may be at risk for getting cancer?	Is everyone at the same risk for getting cancer? Do things like age, gender, ethnic/racial background have an influence?
4	Part of the goal of this project is to understand how people think about cancer and what the needs are so we can better help community members going through this. Would anyone feel comfortable sharing an experience about a family member who either had or currently has cancer?	What was this experience like for your family? What beliefs or views about cancer were discussed as your family member was going through this?
5	We would now like to talk about the risks or chances that people could get cancer. How likely do you think it is that someone could get some type of cancer?	Has anyone heard the term “inherited risk?” What does that mean to you? If someone has cancer in your family, does that make it more likely that someone else will get cancer?
6	Are there things that people do to lower the chances that they could get cancer?	Probe for things like diet, exercise, social, etc.
7	You may have seen information that is available at a place like the health clinic that has information available about someone’s risk of getting cancer. This information might be in a video, written in something like a pamphlet, presented in a group lecture or one-on-one discussion. Which of these would you be interested in as a way to learn about cancer risk?	
8	Are there programs or resources that you would like to see in the community to help people who may have cancer?	
9	Based on our discussion so far, what concerns or worries to do have about cancer that we have not talked about? That is all the questions we have; is there anything further that you would like to talk about or share with us?	

**Table 2:** Description of analysis

Phase	Description of analysis	Product/result
1	Initial review of all six focus group transcripts.	Analysts independently became familiar with focus group questions, facilitator methods, and breadth and nature of participant responses.
2	Brief summary of each question for two focus groups. Analysts deliberately assigned one male and one female focus group and focus groups of different age cohorts in order to review and account for diverse perspectives. Analysts generate initial coding template to iteratively review/refine during team member discussions	Brief summary and observations for each question for all focus groups compiled in one “master spreadsheet” so all analysts were able to review responses side-by-side. Finalized coding template
3	Narrative summary for three questions for all focus groups. Analysts highlight discrete themes and/or categories of responses across focus groups. Analysts present exemplary quotes from focus groups and review with ZHI staff to ensure culturally-relevant interpretations.	Narrative summaries of each question for all focus groups, punctuated with common themes and/or categories. A table of exemplary quotes to complement each narrative. All summaries compiled and presented in a final report (presented here).

**Table 3:** Focus group details

#	Cohort		Date	Participants
	Gender	Age		
1	Women	50+	April 6, 2018	7
2	Men	50+	April 13, 2018	8
3	Women	30–49	April 20, 2018	9
4	Men	30–49	April 27, 2018	8
5	Women	20–29	May 4, 2018	10
6	Men	20–29	May 16, 2018	9
Total	51			
Women	26			
Men	25			
